# Infection in Patients with Decompensated Heart Failure: In-Hospital Mortality and Outcome

**DOI:** 10.5935/abc.20180037

**Published:** 2018-04

**Authors:** Juliano Novaes Cardoso, Carlos Henrique Del Carlo, Mucio Tavares de Oliveira Junior, Marcelo Eidi Ochiai, Roberto Kalil Filho, Antônio Carlos Pereira Barretto

**Affiliations:** Instituto do Coração (InCor) - Faculdade de Medicina da Universidade de São Paulo, São Paulo, SP - Brazil

**Keywords:** Heart Failure / complications, Mortality, Hospitalization, Comorbidity, Lung Diseases / complications, Urinary Tract / physiopathology

## Abstract

**Background:**

Heart failure (HF) is a syndrome, whose advanced forms have a poor prognosis, which is aggravated by the presence of comorbidities.

**Objective:**

We assessed the impact of infection in patients with decompensated HF admitted to a tertiary university-affiliated hospital in the city of São Paulo.

**Methods:**

This study assessed 260 patients consecutively admitted to our unit because of decompensated HF. The presence of infection and other morbidities was assessed, as were in-hospital mortality and outcome after discharge. The chance of death was estimated by univariate logistic regression analysis of the variables studied. The significance level adopted was P < 0.05.

**Results:**

Of the patients studied, 54.2% were of the male sex, and the mean age ± SD was 66.1 ± 12.7 years. During hospitalization, 119 patients (45.8%) had infection: 88 (33.8%) being diagnosed with pulmonary infection and 39 patients (15.0%), with urinary infection. During hospitalization, 56 patients (21.5%) died, and, after discharge, 36 patients (17.6%). During hospitalization, 26.9% of the patients with infection died vs 17% of those without infection (p = 0.05). However, after discharge, mortality was lower in the group that had infection: 11.5% vs 22.2% (p = 0.046).

**Conclusions:**

Infection is a frequent morbidity among patients with HF admitted for compensation of the condition, and those with infection show higher in-hospital mortality. However, those patients who initially had infection and survived had a better outcome after discharge.

## Introduction

Of the cardiovascular diagnoses, heart failure (HF) is the most frequent cause of hospitalization of patients older than 65 years in Brazil and worldwide.^1,2^ Usually HF is controlled at the doctor's office, but when advanced or associated with any disease or comorbidity, the patients can decompensate, requiring hospitalization.^3^ Several factors can contribute to aggravate HF: acute coronary syndrome, arrhythmias and acute respiratory disease were identified as the most common precipitating factors of heart decompensation.^2^ In the OPTIMIZE-HF Registry, acute coronary syndrome and acute respiratory disease were associated with higher in-hospital mortality.^4^ In the emergency department of our hospital, the factors associated with decompensation were non-adherence to treatment, renal failure, arrhythmias and infections.^5^ This study was aimed at analyzing the role of infection in the outcome of patients admitted to our unit, a supporting ward of the emergency department.

## Methods

This is a cohort study assessing 260 patients consecutively admitted to our unit, a supporting ward of the emergency department of the Instituto do Coração (InCor) of the Hospital das Clínicas of the São Paulo University Medical School (HCFMUSP), in 2014 because of decompensated HF. Only the first admission of each patient was considered. All patients had New York Heart Association (NYHA) functional class III or IV HF. They were followed up for up to one year, and underwent clinical, echocardiographic and laboratory assessment.

The following data were assessed: identification, heart disease etiology, comorbidities, clinical findings, such as heart rate and blood pressure at the first assessment, hemodynamic clinical profile, and echocardiographic and laboratory findings. The diagnosis of HF was established by use of the Framingham criteria, and type B natriuretic peptide (BNP), in case of diagnostic doubt, in addition to assessment of ejection fraction by use of two-dimensional echocardiography with color doppler. The comorbidities were identified based on the description of the attending physicians. Renal failure was confirmed by the presence of high levels of urea and creatinine, while diabetes mellitus, by the prescription of hypoglycemic agents on admission. Hypothyroidism was identified in the presence of a prescription of levothyroxine or increased levels of TSH. Atrial fibrillation was diagnosed based on the electrocardiographic tracing, while pulmonary infection was diagnosed based on signs and symptoms, in addition to chest X-ray, blood cell count and C-reactive protein (CRP). Urinary infection was diagnosed based on signs and symptoms, in addition to blood cell count, urinalysis and urine culture. We assessed the characteristics of the patients with infection, and compared them with those of the patients without infection. 

### Statistical analysis

The Kolmogorov-Smirnov test was used to test data normality (p > 0.05 = normal distribution). Regarding the characteristics of the population, continuous variables with normal distribution were presented as mean ± standard deviation. Continuous variables without normal distribution were presented as median (interquartile range 25%-75%). Categorical variables were presented as absolute number and percentage.

When comparing the groups, the continuous variables were presented as mean ± standard deviation. Unpaired Student *t* test was used for variables with normal distribution, and Mann-Whitney *U* test for variables with non-normal distribution. Chi-square test of association or Fisher exact test was used to compare the categorical variables. All tests performed are two-tailed and a p value < 0.05 was considered statistically significant. All statistical analyses were performed with the Statistical Package for the Social Sciences (SPSS) software.

## Results

This study included 260 patients, with a mean age of 66.1 ± 12.7 years, 54.2% of the male sex. The patients were followed up during hospitalization and after discharge. The length of follow-up was 240.05 days (standard error = 10.47, 95% confidence interval = 219.52 - 260.57 days). During hospitalization, 119 patients (45.8%) had infection, 88 (33.8%) being diagnosed with pulmonary infection and 39 patients (15.0%), with urinary infection. Eight patients had both pulmonary^7^ and urinary infections concomitantly. Renal failure was present in 142 patients (54,6%), chronic obstructive pulmonary disease (COPD) in 34 patients (13.1%),^7^ hypothyroidism in 47 patients (18.1%), diabetes mellitus in 95 patients (36.5%), and atrial fibrillation in 119 patients (45.8%). Table 1 shows the major characteristics of the population studied, of which, 170 patients (65.4%) had HF with left ventricular ejection fraction (LVEF) < 40%, 37 (14.2%) had LVEF of 40%-49%, and 53 (20.4%) had LVEF ≥ 50%.

**Table 1:** Characteristics of the population

Characteristics	P (K-S)	N = 260 patients
Age (years)	0.062	66.1 ± 12.7
Male sex - n (%)	-	141 (54.2)
**HF etiology - n (%)**		
Chagasic	-	46 (17.7)
Ischemic	-	97 (37.3)
Non-ischemic, non-Chagasic	-	117 (45.0)
**Comorbidities - n (%)**		
Renal failure	-	142 (54.6)
COPD	-	34 (13.1)
Hypothyroidism	-	47 (18.1)
Diabetes mellitus	-	95 (36.5)
Atrial fibrillation	-	119 (45.8)
Urinary infection	-	39 (15.0)
Pneumonia	-	88 (33.8)
Infection (any site)	-	119 (45.8)
**Vital signs**		
SBP (mm Hg)	< 0.001	100.0 (82.8 - 120.0)
DBP (mm Hg)	< 0.001	60.0 (56.0 - 80.0)
HR (bpm)	0.005	80.0 (70.0 - 98.0)
**Echocardiogram**		
LVDD (mm)	0.523	62.0 ± 10.4
LA (mm)	0.071	48.4 ± 7.2
LVEF (%)	< 0.001	30.0 (25.0 - 45.0)
PAP (mm Hg)	0.392	51.3 ± 15.6
**Hemodynamic profile - admission - n (%)**		
Profile B	-	131 (50.4)
Profile C	-	111 (42.7)
Profile L	-	18 (6.9)
Laboratory tests		
Hemoglobin (g/dl)	0.851	13.1 ± 2.3
Urea (mg/dL)	0.019	79.0 (51.0 - 108.0)
Creatinine (mg/dL)	< 0.001	1.6 (1.2 - 2.0)
Sodium	0.014	138.0 (134.0 - 140.0)
Potassium	0.002	4.3 (4.0 - 4.9)
C-reactive protein	< 0.001	18.0 (7.5 - 53.6)
CKMB mass	< 0.001	2.0 (1.4 - 3.6)
Troponin I	< 0.001	0.05 (0.022 - 0.107)
BNP	< 0.001	1020.0 (457.5 - 2014.3)

P (K-S): teste de Kolmogorov-Smirnov (p > 0.05 = normal distribution). Data are presented as mean ± standard deviation for continuous variables with normal distribution, or median (interquartile range 25% - 75%) for continuous variables with non-normal distribution. Categorical variables are presented as absolute numbers (percentage). COPD: chronic obstructive pulmonary disease; SBP: systolic blood pressure; DBP: diastolic blood pressure; HR: heart rate; LVDD: left ventricular diastolic diameter; LA: left atrium; LVEF: left ventricular ejection fraction; PAP: pulmonary artery pressure.

The mean length of stay was 28.6 days (20.52). During hospitalization, 56 patients (21.5%) died. Within 30 days from discharge, 58 patients (28.43%) required a visit to the emergency department, and 28 (13.73%), a new admission.

When comparing the groups with and without infection, the following characteristics were similar: age, sex, hemoglobin, blood pressure, heart rate and LVEF (Table 2). Renal failure was present in 73 patients (61.3%) with infection vs 69 patients (48.9%) without infection (P = 0.045). The mean dose of furosemide was similar in both groups: 68.06 mg/day (37.58) for the infected patients vs 71.84 mg/day (39.23) for non-infected ones (P = 0.568). In the group with infection, 42 patients (35.3%) died during the total follow-up vs 50 patients (35.5%) of the group without infection (P = 0.977). During hospitalization, 32 patients with infection (26.9%) died vs 24 patients without infection (17%) (P = 0.054). When assessing only the discharged patients, 10 of the group with infection (11.5%) died during follow-up vs 26 (22.2%) of the group without infection (P = 0.047).

**Table 2:** Comparison of the characteristics of the patients with and without infection

Characteristics	Infection		p*
	Yes (n = 119)	No (n = 141)	
Age (years)	67.33 ± 12.18	65.03 ± 13.04	0.147
Male sex - n (%)	58 (48.7)	83(58.9)	0.102
Hemoglobin (g/dl)	12.93 ± 1.93	13.25 ± 2.47	0.251
SBP (mm Hg)	100.0 (83.5 - 123.5)	96 (81.5 - 120.0)	0.109
DBP (mm Hg)	61.0 (53.0 - 80.0)	60.0 (56.0 - 76.0)	0.701
HR (bpm)	84.0 (70.0 - 100.0)	80.0 (67.8 - 94.5)	0.493
LVEF (%)	30.0 (25.0 - 46.0)	30 (25.0 - 45.0)	0.019
LVDD (mm)	60.60 ± 10.07	63.24 ± 10.46	0.044
LVSD (mm)	48.72 ± 12.52	52.45 ± 12.74	0.022
Urea (mg/dL)	78.0 (56.0 - 107.0)	79.0 (49.3 - 108.0)	0.391
Creatinine (mg/dL)	1.62 (1.23 - 2.17)	1.54 (1.22 - 2.00)	0.680
Renal failure - n (%)	73 (61.3)	69 (48.9)	0.045
Length of stay (days)	29.43 ± 19.43	21.3 ± 27.89	0.546
**Mortality - n (%)**			
Total	42 (35.3)	50 (35.5)	0.977
In-hospital	32 (26.9)	24 (17)	0.050
Post-discharge	10 (11.5%)	26 (22.2%)	0.046

Data are presented as mean ± standard deviation for continuous variables with normal distribution, or median (interquartile range 25% - 75%) for continuous variables with non-normal distribution. Categorical variables are presented as absolute numbers (percentage). P*: To calculate P value, Student t test was used for the variables with normal distribution, Mann-Whitney U test for the variables with non-normal distribution. P value was estimated by use of the chi-square test or Fisher exact test for the categorical variables. SBP: systolic blood pressure; DBP: diastolic blood pressure; HR: heart rate; LVDD: left ventricular diastolic diameter; LVSD: left ventricular systolic diameter; LVEF: left ventricular ejection fraction.

Table 3 shows the characteristics related to in-hospital mortality, and Table 4 shows mortality during total follow-up. Renal failure was observed in 54.6% of the patients, more frequently in those who died during hospitalization (Table 3) or during the total study period (Table 4).

**Table 3:** Comparison of the characteristics of the patients regarding in-hospital mortality

Characteristics	In-Hospital Death		p*
	Yes (n =56)	No (n = 204)	
Age (years)	65.7 ± 12.6	66.2 ± 12.8	0.817
Male sex - n (%)	33 (58.9)	108 (52.9)	0.426
**HF etiology - n (%)**			
Chagasic	11 (19.6)	35 (17.2)	0.666
Ischemic	21 (37.5)	76 (37.3)	0.973
**Comorbidities - n (%)**			
Renal failure	43 (76.8)	99 (48.5)	< 0.001
COPD	7 (12.5)	27 (13.2)	0.885
Hypothyroidism	11 (19.6)	36 (17.6)	0.731
Diabetes mellitus	18 (32.1)	77 (37.7)	0.441
Atrial fibrillation	28 (50.0)	91 (44.6)	0.473
Urinary infection	10 (17.9)	29 (14.2)	0.499
Pneumonia	24 (42.9)	64 (31.4)	0.108
Infection (any site)	32 (57.1)	87 (42.6)	0.054
**Vital signs**			
SBP (mm Hg)	91 (80-110)	100 (84-120)	0.109
DBP (mm Hg)	60 (58.5-77)	61.5 (55.25-80)	0.701
HR (bpm)	79 (62-98)	80 (70-97.75)	0.493
**Echocardiogram**			
LVDD (mm)	64.6 ± 9.4	61.4 ± 10.6	0.043
LVSD (mm)	53.88 ±10.95	49.93 ± 13.12	0.050
LA (mm)	48.7 ± 8.0	48.4 ± 7.0	0.746
LVEF (%)	28 (24.25-35)	32 (25-47)	0.019
PAP (mm Hg)	55.7 ± 16.4	50.1 ± 15.2	0.035
**Hemodynamic profile - admission - n (%)**			
Profile B	23 (41.1)	108 (52.9)	0.116
Profile C	30 (53.6)	81 (39.7)	0.063
Profile L	3 (5.4)	15 (7.4)	0.771
**Laboratory tests**			
Hemoglobin	12.3 ± 2.1	13.3 ± 2.2	0.004
Urea (mg/dL)	83 (55-114)	74.5 (51-107)	0.391
Creatinine (mg/dL)	1.66 (1.09-2)	1.55 (1.23-2.06)	0.680
Sodium	137 (133-140)	138 (135-140)	0.598
Potassium	4.3 (4-5)	4.3 (4-4.8)	0.583
C-reactive protein	19.44 (8.82-50.2)	18 (7.39-54)	0.766
CKMB mass	1.94 (1.24-3.64)	2.03 (1.41-3.58)	0.950
Troponin I	0.05 (0.02-0.12)	0.05 (0.02-0.10)	0.951
BNP	1283 (853-2095)	969 (422.5-1951.5)	0.101
Total cholesterol	134.9 ± 44.7	145.8 ± 46.0	0.308
HDL	30.5 ± 14.9	38.3 ± 15.0	0.015
LDL	82.4 ± 36.1	86.6 ± 36.3	0.504
**Vasoactive drugs on admission - n (%)**			
Dobutamine	48 (85.7)	111 (54.4)	< 0.001
Levosimendan	2 (3.6)	16 (7.8)	0.378
Milrinone	5 (8.9)	4 (2.0)	0.024

Data are presented as mean ± standard deviation for continuous variables with normal distribution, or median (interquartile range 25% - 75%) for continuous variables with non-normal distribution. Categorical variables are presented as absolute numbers (percentage). P*: To calculate P value, Student t test was used for the variables with normal distribution, Mann-Whitney U test for the variables with non-normal distribution. P value was estimated by use of the chi-square test or Fisher exact test for the categorical variables. COPD: chronic obstructive pulmonary disease; SBP: systolic blood pressure; DBP: diastolic blood pressure; HR: heart rate; LVDD: left ventricular diastolic diameter; LVSD: left ventricular systolic diameter; LA: left atrium; LVEF: left ventricular ejection fraction; PAP: pulmonary artery pressure.

**Table 4:** Comparison of the characteristics of the patients regarding total mortality (in hospital + post-discharge)

Characteristics	Total Death		p*
	Yes (n = 92)	No (n = 168)	
Age (years)	65.9 ± 12.4	66.2 ± 12.9	0.828
Male sex - n (%)	55 (59.8)	86 (51.2)	0.184
**HF etiology - n (%)**			
Chagasic	21 (22.8)	25 (14.9)	0.108
Ischemic	31 (33.7)	66 (39.3)	0.373
**Comorbidities - n (%)**			
Renal failure	64 (69.6)	78 (46.4)	<0.001
COPD	10 (10.9)	24 (14.3)	0.435
Hypothyroidism	17 (18.5)	30 (17.9)	0.901
Diabetes mellitus	28 (30.4)	67 (39.9)	0.130
Atrial fibrillation	47 (51.1)	72 (42.9)	0.203
Urinary infection	14 (15.2)	25 (14.9)	0.942
Pneumonia	31 (33.7)	57 (33.9)	0.970
Infection (urinary and/or pulmonary)	42 (45.7)	77 (45.8)	0.978
**Vital signs:**			
SBP (mm Hg)	91 (84-110)	100 (82-125)	0.023
DBP (mm Hg)	60 (57.5-70)	63.5 (55-80)	0.465
HR (bpm)	80 (69.5-100)	80 (70-94.5)	0.898
**Echocardiogram:**			
LVDD (mm)	64.0 ± 9.4	61.0 ± 10.8	0.029
LA (mm)	48.3 ± 7.3	48.5 ± 7.1	0.859
LVEF (%)	28.5 (24.25-35)	35 (25-49)	0.002
PAP (mm Hg)	54.2 ± 15.8	49.8 ± 15.3	0.058
**Hemodynamic profile - admission - n (%)**			
Profile B	37 (40.2)	94 (56.0)	0.015
Profile C	50 (54.3)	61 (36.3)	0.005
Profile L	5 (5.4)	13 (7.7)	0.484
**Laboratory tests**			
Hemoglobin	12.4 ± 2.1	13.5 ± 2.3	< 0.001
Urea (mg/dL)	82 (54-114)	74.5 (51-107)	0.453
Creatinine (mg/dL)	1.66 (1.23-2)	1.54 (1.22-2.03)	0.481
Sodium	137 (134-139)	138 (135-140)	0.325
Potassium	4.3 (4-4.9)	4.4 (4-4.8)	0.835
C-reactive protein	17.97 (8.94-48.8)	18.88 (7.31-60.42)	0.927
CKMB mass	1.98 (1.32-3.26)	2.1 (1.5-3.6)	0.759
Troponin I	0.05 (0.02-0.11)	0.05 (0.02-0.1)	0.941
BNP	1274 (774-2095)	969 (383.5-1951.5)	0.098
Total cholesterol	138.0 ± 44.9	147.0 ± 46.2	0.306
HDL	32.6 ± 15.0	39.1 ± 15.0	0.013
LDL	83.5 ± 35.8	87.1 ± 36.5	0.499
**Vasoactive drugs on admission - n (%)**			
Dobutamine	72 (78.3)	87 (51.8)	< 0.001
Levosimendan	6 (6.5)	12 (7.1)	0.850
Milrinone	5 (5.4)	4 (2.4)	0.286

Data are presented as mean ± standard deviation for continuous variables with normal distribution, or median (interquartile range 25% - 75%) for continuous variables with non-normal distribution. Categorical variables are presented as absolute numbers (percentage). P*: To calculate P value, Student t test was used for the variables with normal distribution, Mann-Whitney U test for the variables with non-normal distribution. P value was estimated by use of the chi-square test or Fisher exact test for the categorical variables. COPD: chronic obstructive pulmonary disease; SBP: systolic blood pressure; DBP: diastolic blood pressure; HR: heart rate; LVDD: left ventricular diastolic diameter; LA: left atrium; LVEF: left ventricular ejection fraction; PAP: pulmonary artery pressure.

## Discussion

Infection was associated with decompensated HF in 45.8% of the patients, and in that group of infected patients, an increase in mortality was observed during hospitalization. However, after hospital discharge, the group with infection showed better outcome as compared to those without infection. The most frequent comorbidity in our study was renal failure, affecting 54.6% of the patients, and relating to in-hospital mortality during follow-up after discharge.

The causes of heart decompensation varied according to the population studied. Acute coronary syndrome, arrhythmias and acute respiratory disease are the most frequent precipitating factors of heart decompensation.^2^ At the emergency department of our hospital, the most common cause of hospitalization was non-adherence to treatment, and infections were considered the cause of hospitalization in 8% of the cases.^5^ In the BREATHE Registry, poor adherence was also the most frequent cause, and infection was the second one, contributing to decompensation in 22.9% of the cases.^6^ The association between infection and decompensation and worse prognosis is well known. An investigation performed at the InCor, via the statistics department, showed that, in the last 10 years, 27,528 patients were hospitalized and diagnosed with HF (I50), most of the male sex (55%). The mean length of stay was 14.8 days, and the in-hospital mortality of that population with HF was 24.8%.^6^

In the present study, of the patients admitted to our ward in 2014, the in-hospital mortality of those with HF and infection was 26.9% versus 17.0% of those without infection (p = 0.05). The increase in mortality due to infection has been also reported in the OPTIMIZE-HF Registry.^4^

Comparing the characteristics of the patients with and without infection based on the variables analyzed, those with infection decompensated with a milder ventricular impairment than that of those without infection, suggesting that decompensation resulted from the overload and systemic changes that infection causes and not only from the severity of cardiac impairment. Patients with infection had smaller heart dilatation than those without infection, 60.6 mm (10.07) vs 63.4 mm (10.46), p = 0.04. After hospital discharge, patients of the group with infection had better outcome. Mortality during follow-up of those who were hospitalized with infection was 11.5% versus 22.2% of those without infection (p = 0.046). That lower mortality can be attributed to the milder cardiac impairment of those who had infection, a fact that can explain the best outcome after discharge with infection under control. This result shows that infection worsens the prognosis of patients who, even without significant cardiac impairment, decompensate and have a tendency towards worse outcome during hospitalization. Our data confirm that infection worsens the outcome of patients with HF.

In addition, in their study, M. Arrigo et al. have also reported similar findings for patients with infection, as well as lower re-hospitalization rates as compared to those of patients without in-hospital infection.^2^ Those data show that infections, by overloading impaired hearts, worsen the clinical findings, leading to decompensation, and such patients with impaired hearts have a worse outcome than those without infection. Once infection is under control, the milder heart impairment of those patients determines their better outcome as compared to those who decompensated without infection, because of their more severe heart impairment.

Those findings emphasize the importance of pulmonary infection prevention, avoiding the worsening of patients and their consequent hospitalization. That benefit has been confirmed in octogenarian patients, and those who received vaccination were less frequently hospitalized.^8^ Pneumococcal and influenza vaccinations as recommended in our guideline might be very useful to prevent pulmonary infection.

### Limitations

This is an observation study, with its inherent limitations. The patients were selected from those admitted to a tertiary hospital, which might determine the bias of more severe cases.

## Conclusions

Infection is a frequent morbidity among patients with HF admitted for compensation of the condition, and those with infection show higher in-hospital mortality. However, those patients who initially had infection and survived had a better outcome after discharge.

## References

[1] Ponikowski P, Voors AA, Anker SD, Bueno H, Cleland JG, Coats AJ (2016). 2016 ESC Guidelines for the diagnosis and treatment of acute and
chronic heart failure: the Task Force for the diagnosis and treatment of
acute and chronic heart failure of the European Society of Cardiology (ESC)
Developed with the special contribution of the Heart Failure Association
(HFA) of the ESC. Eur Heart J.

[2] Brasil, Ministério da Saúde Datasus: mortalidade - 1996 a 2015, pela CID-10.

[3] Arrigo M, Tolppanen H, Sadoune M, Feliot E, Teixeira A, Laribi S (2016). Effect of precipitating factors of acute heart failure on
readmission and long-term mortality. ESC Heart Fail.

[4] Fonarow GC, Abraham WT, Albert NM, Stough WG, Gheorghiade M, Greenberg BH, OPTIMIZE-HF Investigators and Hospitals (2008). Factors identified as precipitating hospital admissions for heart
failure and clinical outcomes: findings from OPTIMIZE-HF. Arch Intern Med.

[5] Mangini S, Silveira FS, Silva CP, Grativvol PS, Seguro LF, Ferreira SM (2008). Decompensated heart failure in the emergency department of a
cardiology hospital. Arq Bras Cardiol.

[6] Albuquerque DC, Souza Neto JD, Bacal F, Rohde LE, Pereira SB, Berwanger O, Investigadores Estudo BREATHE (2015). I Registro Brasileiro de Insuficiência Cardíaca:
aspectos clínicos, qualidade assistencial e desfechos
hospitalares. Arq Bras Cardiol.

[7] Pereira-Barretto AC, Cardoso JN, Del Carlo CH, Ramos Neto JA, Kalil Filho R Pneumonia em pacientes com Insuficiência Cardíaca aumenta
a mortalidade durante hospitalização.

[8] Ahmed MB, Patel K, Fonarow GC, Morgan CJ, Butler J, Bittner V (2016). Higher risk for incident heart failure and cardiovascular
mortality among community-dwelling octogenarians without pneumococcal
vaccination. ESC Heart Fail.

